# Inhibition of Wnt/beta-catenin signaling downregulates expression of aldehyde dehydrogenase isoform 3A1 (ALDH3A1) to reduce resistance against temozolomide in glioblastoma *in vitro*

**DOI:** 10.18632/oncotarget.25210

**Published:** 2018-04-27

**Authors:** Abigail Kora Suwala, Katharina Koch, Dayana Herrera Rios, Philippe Aretz, Constanze Uhlmann, Isabella Ogorek, Jörg Felsberg, Guido Reifenberger, Karl Köhrer, René Deenen, Hans-Jakob Steiger, Ulf D. Kahlert, Jaroslaw Maciaczyk

**Affiliations:** ^1^ Department of Neurosurgery, University Hospital Düsseldorf, Düsseldorf, Germany; ^2^ Department of Neuropathology, University Hospital Düsseldorf, Düsseldorf, Germany; ^3^ German Cancer Consortium (DKTK), Partner Site Essen/Düsseldorf, German Cancer Research Center (DKFZ), Heidelberg, Germany; ^4^ Genomics and Transcriptomics Laboratory, Biological and Medical Research Center (BMFZ), Heinrich Heine University, Düsseldorf, Germany; ^5^ Department of Surgical Sciences-Neurosurgery, Dunedin School of Medicine, University of Otago, Dunedin, New Zealand

**Keywords:** ALDH3A1, glioma, Wnt, chemoresistance, temozolomide

## Abstract

Glioblastoma is the most aggressive type of glioma. The Wingless (Wnt) signaling pathway has been shown to promote stem cell properties and resistance to radio- and chemotherapy in glioblastoma. Here, we demonstrate that pharmacological Wnt pathway inhibition using the porcupine inhibitor LGK974 acts synergistically with temozolomide (TMZ), the chemotherapeutic drug currently used as standard treatment for glioblastoma, to suppress *in vitro* growth of glioma cells. Synergistic growth inhibition was independent of the *O*^6^-alkylguanine DNA alkyltransferase (*MGMT*) promoter methylation status. Transcriptomic analysis revealed that expression of aldehyde dehydrogenase 3A1 (*ALDH3A1*) was significantly down-regulated when cells were treated with LGK974 and TMZ. Suppressing ALDH3A1 expression increased the efficacy of TMZ and reduced clonogenic potential accompanied by decreased expression of stem cell markers CD133, Nestin and Sox2. Taken together, our study suggests that previous observations concerning Wnt signaling blockade to reduce chemoresistance in glioblastoma is at least in part mediated by inhibition of ALDH3A1.

## INTRODUCTION

Glioblastoma is the most common primary malignant brain tumor in adults and is characterized by a dismal prognosis. Despite radical treatment with radio- and chemotherapy, the median overall survival is less than two years [1, 2]. One of the obstacles of curative treatment of glioblastoma is primary or acquired resistance to the current standard of care consisting of radiotherapy and chemotherapy with temozolomide (TMZ). In case of TMZ, which works as a DNA alkylating agent by adding alkyl-residues to the N-7 and O-6 positions of guanine, promoter methylation of the O-6-methylguanine-DNA methyltransferase (*MGMT*) gene is associated with more efficient therapeutic success [3]. TMZ is less effective in glioblastomas lacking *MGMT* promoter methylation, resulting in worse outcome of this group of patients [4]. New therapeutic options, such as anti-angiogenic strategies employing blocking antibodies against vascular endothelial growth factor, did not result in significant overall survival benefit [5]. Therefore, other treatment targets must be identified and validated, with glioma stem-like cells (GSCs) having emerged as a promising target for future treatment. GSCs have been reported to be the most therapy resistant type of tumor cells in malignant gliomas, withstanding treatments with radio- and chemotherapy [6, 7]. Similar to somatic stem cells, GSCs are governed by deregulated phylogenetically conserved stem cell signaling pathways modulating differentiation, proliferation, invasion and stress regeneration capability [8]. One of these cascades is the Wingless (Wnt) pathway. Several drugs targeting various members of the Wnt signaling network have been developed and have shown promising preclinical results against various types of cancer including glioblastoma [9–13]. We have previously shown that pharmacological interference with the Wnt ligand-receptor interaction through inhibition of porcupine with LGK974 (Wnt974) is a novel strategy to efficiently block Wnt signaling activity in glioblastoma cells [14]. Here, we now demonstrate that LGK974 acts synergistically with TMZ chemotherapy to reduce cell viability in both *MGMT* promoter-methylated and unmethylated glioblastoma neurosphere cell lines. We found aldehyde dehydrogenase 3A1 (ALDH3A1), an enzyme involved in cellular metabolic clearance and detoxification of alcohol-derived acetaldehyde [15], to be down-regulated in cells treated with LGK974 and TMZ as compared to monotherapy with either TMZ or LGK974. Our results suggest that ALDH3A1 in glioblastoma is a target gene of the canonical Wnt signaling and we provide functional evidence that pharmacological inhibition of the pathway by porcupine inhibition increases susceptibly to TMZ treatment at least in part due to down-regulation of ALDH3A1. Therefore, targeting ALDH3A1 can be an innovative strategy to increase TMZ sensitivity in brain cancer cells independently of the *MGMT* promoter methylation status.

## RESULTS

### Pharmacological Wnt inhibition acts synergistically with TMZ to inhibit glioma cell growth

**Figure 1:**
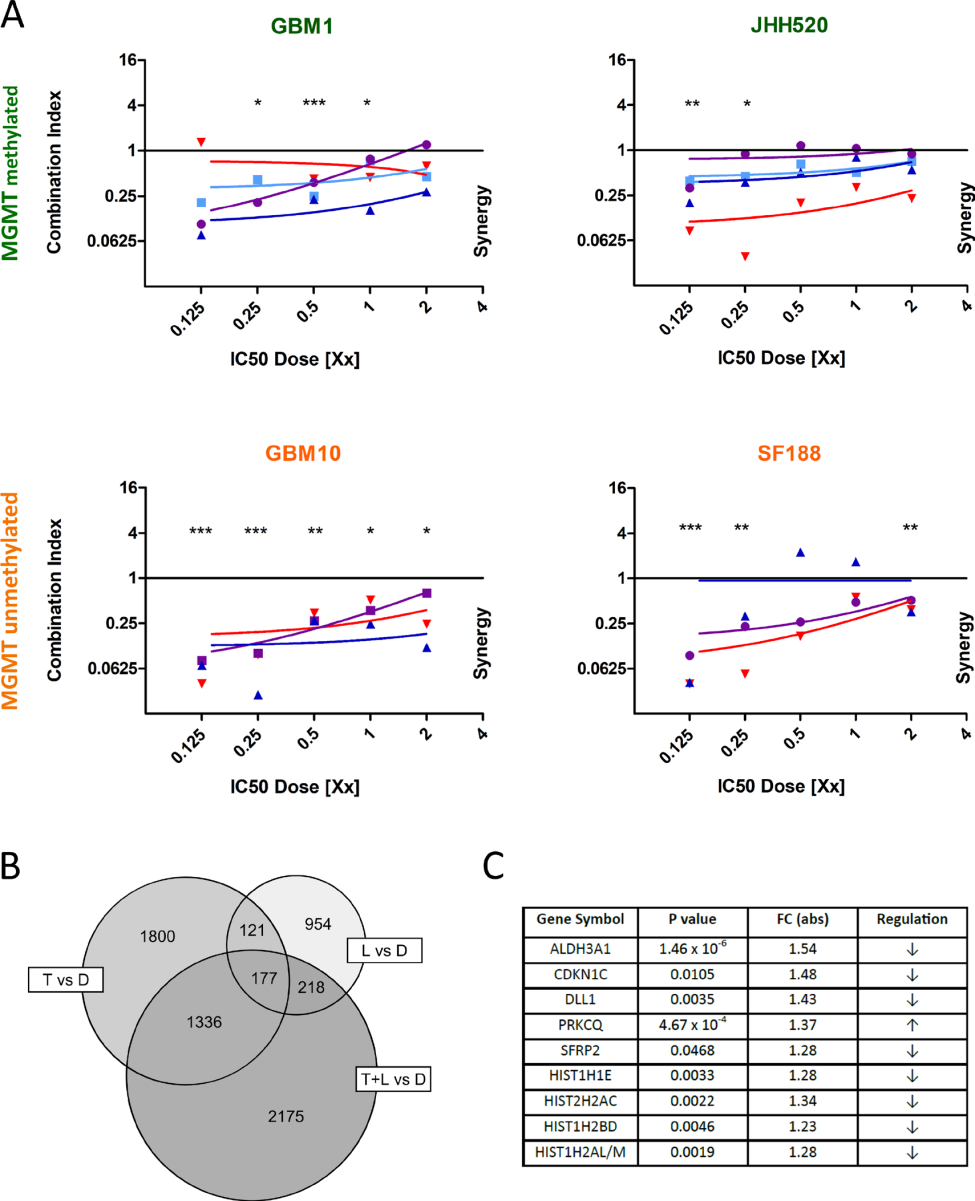
LGK974 acts synergistically in combination with TMZ The x-axis represents different multiples of the detected IC_50_ dose for each drug and cell line. The axis of ordinates demonstrates the calculated combination index. The combination index is calculated based on the median-effect equation, taking each value from TMZ single treatment, LGK974 single treatment and combination of both treatments for one specific dose into account. In one experiment, the combination index is calculated for five different doses and represented by one colored line. If the combination index is less than 1, both treatments act synergistically. If it is equal 1, both treatments act additively. If the combination index is more than 1, the effects are antagonistic. (**A**) LGK974 acts synergistically with TMZ in cell lines with methylated (green labeled GBM1 and JHH520) (*n* = 4 independent experiments) and unmethylated *MGMT* promoter (orange labeled GBM10 and SF188) (*n* = 3 independent experiments). (**B**) 2175 genes are significantly deregulated when combining LGK974 and TMZ treatment in GBM1 cells. Each treated group (TMZ alone, LGK974 alone, TMZ plus LGK974) was compared to the DMSO-treated control group (*n* = 4 independent experiments). 1800 genes were differentially expressed in the TMZ-treated group, 954 genes in the LGK974-treated group and 177 genes were expressed in all treated groups compared to DMSO control. (**C**) The nine genes with highest fold-change in the combinatory treatment group. Abbr.: D: DMSO; T: TMZ; L: LGK974.^*^p < 0.05, ^**^p < 0.01, ^***^p < 0.001 vs. additive effect (unpaired student *t*-test).

We characterized the *MGMT* promoter methylation status of four *in vitro* glioma models using methylation-specific PCR as either *MGMT* promoter-methylated (GBM1, JHH520) or -unmethylated (GBM10, SF188) supported by relatively low (GBM1, JHH520) or high (GBM10, SF188) IC_50_ values of TMZ (Table 1). IC_50_ concentrations for LGK974 and doses for γ-radiation were also determined for each cell line. In contrast to TMZ treatment, the *MGMT* promoter methylation status had no effect on therapy sensitivity when LGK974 or γ-radiation was applied to the cultures (Table 1). We previously showed that LGK974 effectively blocks canonical Wnt signaling activity in glioblastoma cells [14]. To assess whether LGK974 might increase sensitivity towards TMZ, we measured combinatory effects of both drugs by defining cell viability (using CellTiter Blue) as our primary readout and calculating synergistic effects due to combination index equation for multiple drug effect interactions using computerized simulations (Compusyn) [16]. We found that the combination of TMZ with LGK974 reduced cell growth significantly more effectively as compared to treatments with either drug alone irrespective on the *MGMT* promoter-methylation status (Figure 1A). The same effect was observed in cells treated with γ-radiation and LGK974 (Supplementary Figure 1A). Looking at the dose-effect curves for both drugs, we observed a sigmoidal curve for TMZ and a hyperbolic, non-linear relationship for LGK974 that explains diminishing increment of effectiveness as the concentration rises above the IC_50_ [17] (Supplementary Figure 1B). Stronger synergy was noticed in lower dosages of both drugs.

**Table 1:** IC_50_ doses for TMZ, LGK974 and γ-irradiation

Cell line	MGMT promoter	LGK974 [μM]	TMZ [μM]	γ-irradiation [Gy]
GBM1	methylated	1	5	2
JHH520	methylated	3	10	6
GBM10	unmethylated	6	70	2
SF188	unmethylated	1	40	2

Each cell line was treated with different dosages of TMZ and LGK974. Cell viability was taken as readout.

### Base line ALDH3A1 expression is independent on Wnt pathway activity and TMZ resistance but is down-regulated upon treatment with LGK974 and TMZ

**Figure 2:**
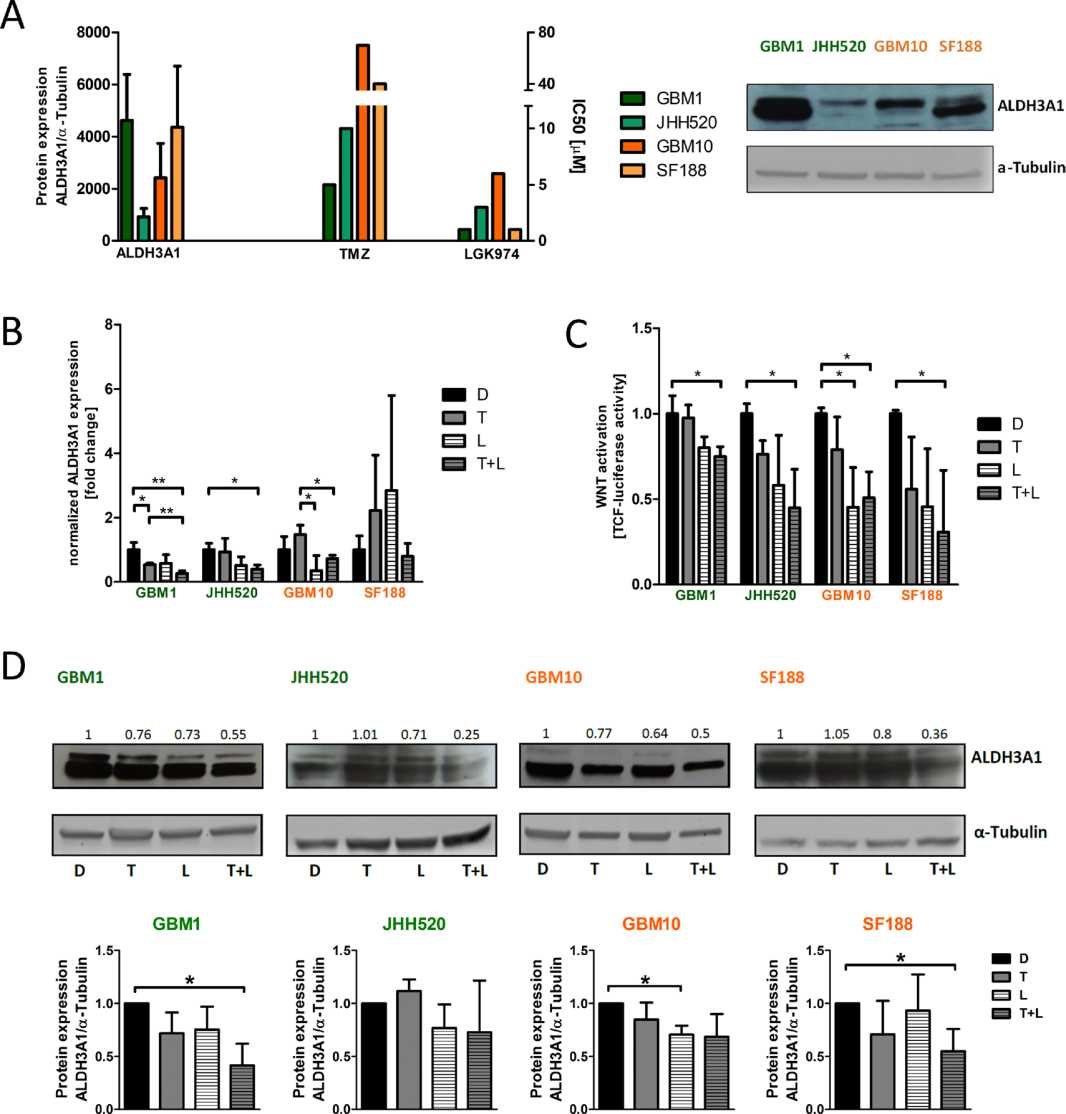
ALDH3A1 mRNA and protein expression is down-regulated in glioma cells treated with LGK974 and TMZ (**A**) Comparison between basal protein levels of ALDH3A1 (left) and IC_50_ dosages of TMZ and LGK974 (right) for all four cell lines. One blot of basal ALDH3A1 expression is shown (*n* = 3 individual experiments). (**B**) *ALDH3A1* mRNA expression in the four glioma cell lines. Note that *ALDH3A1* mRNA expression is significantly down-regulated in the *MGMT* promoter-methylated cell lines GBM1 and JHH520 in the LGK974 plus TMZ treatment group (*n* = 3 independent experiments). (**C**) Wnt pathway activity assessed by T cell factor (TCF) luciferase reporters. Wnt activity is significantly reduced upon combined treatment with TMZ and LGK974 in all cell lines (*n* = 3 independent experiments). (**D**) Protein expression of ALDH3A1 in the investigated four glioma cell lines upon treatment. Quantification of blots from *n* = 3 independent experiments reveal, ALDH3A1 protein expression is down-regulated in the LGK974 plus TMZ treatment group in GBM1 and SF188. GBM10 showed significant suppression of ALDH3A1 when treated with LGK974 alone, JHH520 did not reduce ALDH3A1 expression. MGMT methylated cell lines presented in green, MGMT unmethylated cell lines presented in orange. Data are presented as mean ± standard deviation (SD). ^*^p < 0.05 (unpaired student *t*-test). D, DMSO; T, TMZ; L, LGK974.

Next we performed the whole transcriptome analysis of GBM1 cells treated with DMSO control, TMZ, LGK974, and TMZ plus LGK974. We chose GBM1 for our analysis as we previously showed that LGK974 effectively reduces stemness in this cell line [14]. Evaluation of the data revealed significant down- or upregulation of 2175 genes with moderate fold change values in the combinatory treatment group as compared to the single treatment groups and the DMSO control group (Figure 1B). We validated the data by targeted expression analyses using quantitative real time PCR (qPCR) for nine differentially expressed genes. The nine genes were selected based on their relatively high overall expression values and the fold-change values of differential expression following combinatory treatment (Figure 1C, Supplementary Figure 2A). All genes showed similar expression tendencies as detected in the microarray screen, except HIST1H2BD, that showed an upregulation on the mRNA expression level (Supplementary Figure 2A). Thereby we confirmed that *ALDH3A1*, the strongest inhibited gene in the microarray screen, was robustly down-regulated by LGK974 and TMZ in GBM1 cells evaluated by real time-PCR. Moreover, we tested basal mRNA and protein expression of *ALDH3A1* of our cell lines and compared it with the specific IC_50_ dosage of TMZ (Figure 2A, Supplementary Figure 2B). We did not find any correlation between ALDH3A1 expression and resistance against TMZ, which is more predicted by MGMT promoter methylation status. There was also no correlation found between ALDH3A1 expression and basal WNT activity in our cell lines (Supplementary Figure 2C).

Testing of *ALDH3A1* mRNA expression after pharmacological treatment in additional cell models SF188, JHH520 and GBM10 revealed that the latter two showed significant signal suppression. No difference in gene transcription was noticed in pediatric glioma model SF188 (Figure 2B). For these studies we used half dosages of the IC_50_ for each drug, since these concentrations resulted in most significant synergistic effects (Figure 1A). The array data suggested that LGK974 treatment alone may not only down-regulate specific Wnt target genes such as Dickkopf3 and CD44, but also down-regulates expression of *ALDH3A1,* although to lesser extent as compared to combination treatment with TMZ (data not shown). Verifying this data using sensitive reporter readouts, we found combined TMZ and LGK974 treatment significantly reducing Wnt pathway activity in all four cell lines (Figure 2C). On protein level, combination treatment of LGK974 and TMZ reduced ALDH3A1 in GBM1 and SF188, whereas in GBM10 protein was reduced under LGK974 monotherapy only. No significant suppression of ALDH3A1 was seen in JHH520 (Figure 2D). In concordance, genetic inhibition of Wnt signaling in GBM1 cells using shRNA-mediated knock-down of β-catenin expression caused reduction in ALDH3A1 expression levels (Supplementary Figure 2D).

### ALDH3A1 inhibition reduces cell viability and resistance to TMZ

**Figure 3:**
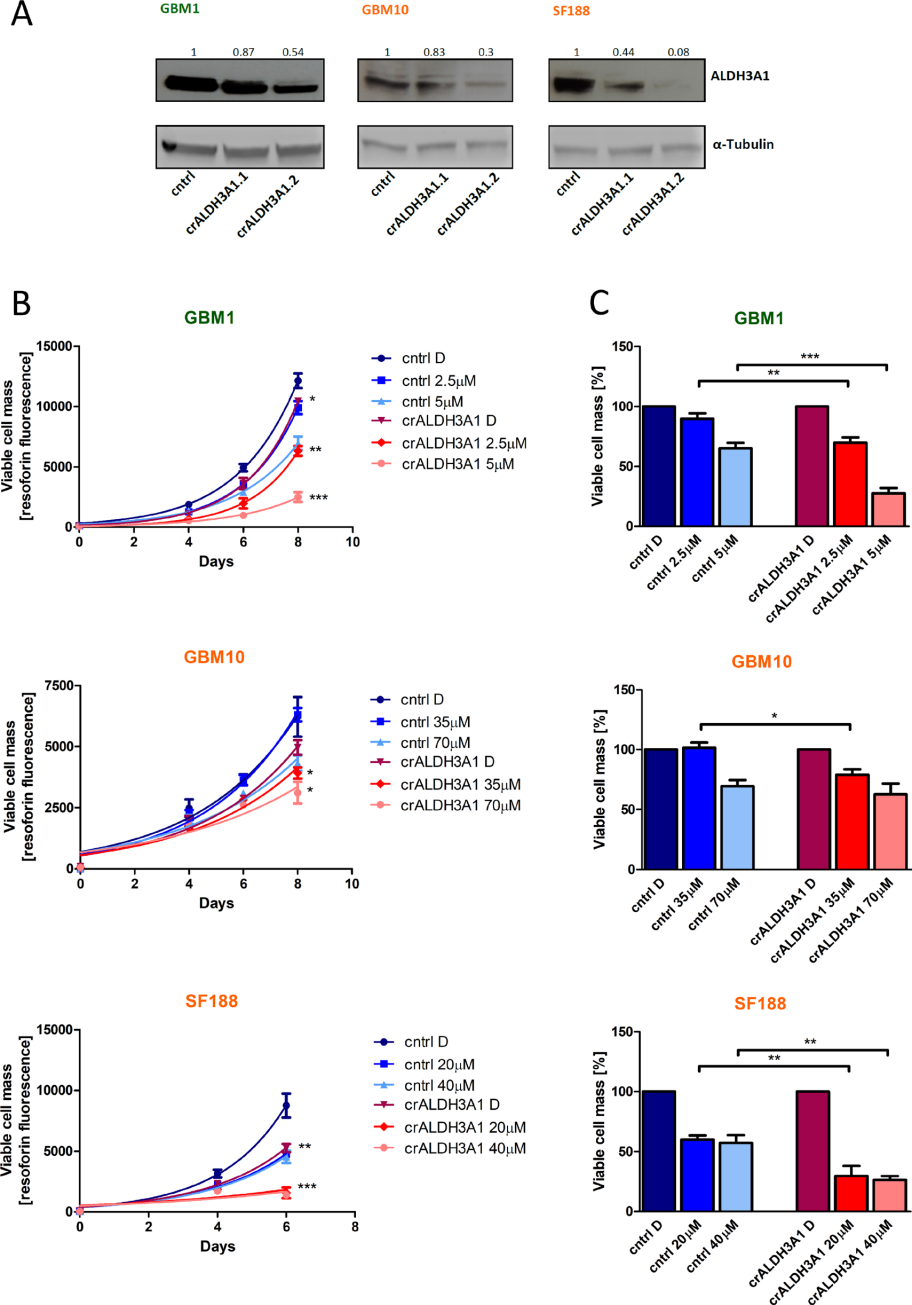
*ALDH3A1* knock-down reduces cell viability and sensitizes cells to TMZ (**A**) Two individual knock-downs of ALDH3A1 were established in three cell lines. α-Tubulin served as gel loading control. (**B**) *ALDH3A1* knock-down (crALDH3A1.2) and control cells were treated with two different concentrations of TMZ, based on the IC_50_ of the control cells (1 × IC_50_, 0.5 × IC_50_). Cell viability was measured at day 0, 4, 6 (and 8 in slowly growing cells). Statistical evaluation was performed for *ALDH3A1* knock-down cells compared to control cells treated with DMSO or the same concentration of TMZ. (**C**) Cell viability of the DMSO-treated cells of either ALDH3A1 knock-down or control cells is normalized to 100%. ^*^p < 0.05, ^**^p < 0.01, ^***^p < 0.001 (unpaired student *t*-test). D: DMSO.

**Figure 4:**
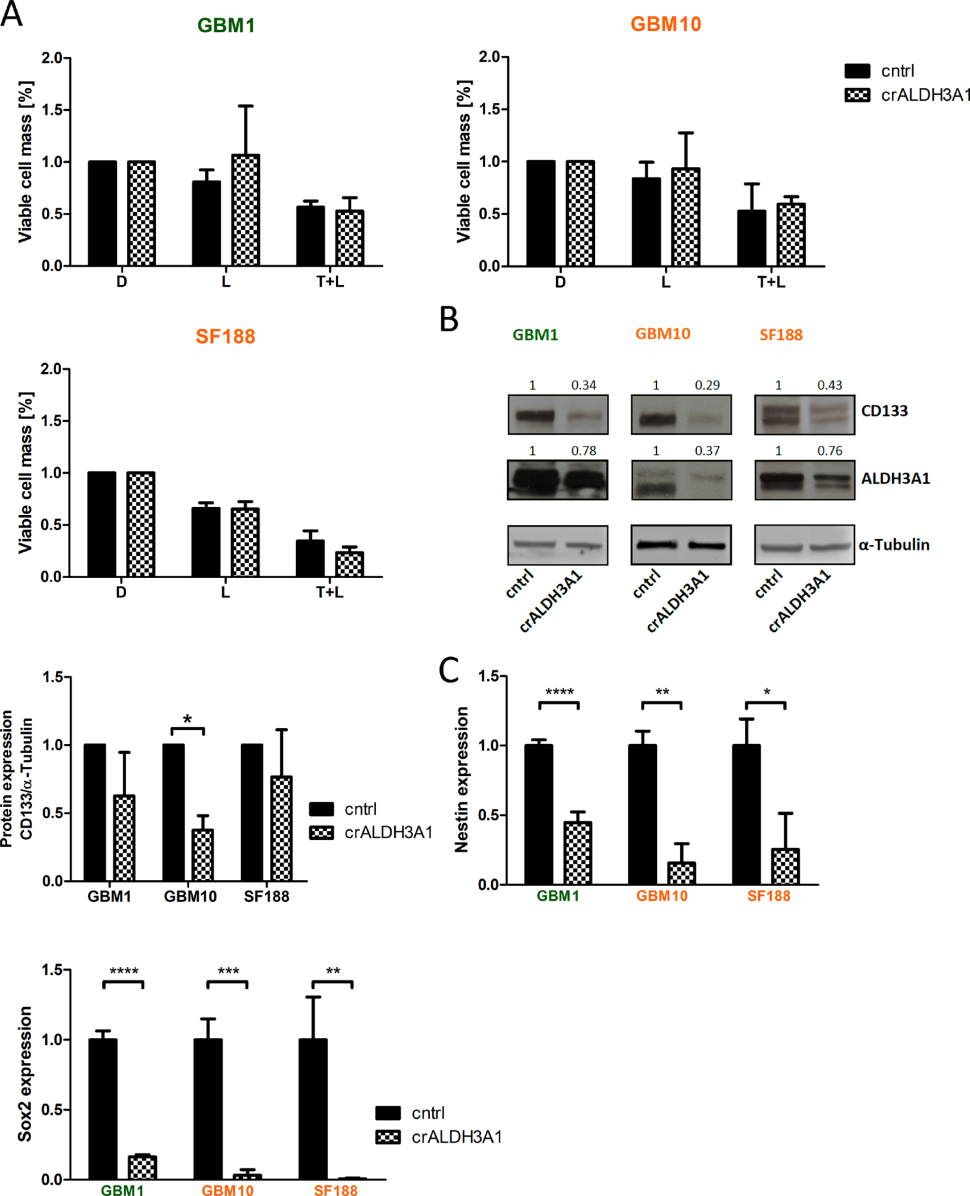
*ALDH3A1* knock-down is not effected by Wnt-inhibition and shows reduced stemness properties (**A**) *ALDH3A1* knock-down (crALDH3A1.2) cells show similar response to LGK974 and combination with TMZ treatment as control cells. Cell viability was measured at day 6. Cell viability of the DMSO-treated cells of either ALDH3A1 knock-down or control cells is normalized to 100% (*n* = 3 independent experiments). (**B**) Protein expression of CD133 in GBM10 is strongly reduced when targeting ALDH3A1 as compared to control cells. In GBM1 and SF188 a tendency of CD133 was noticed. Quantifications of three independent experiments are shown. (**C**) mRNA expression of stemness factors Nestin and Sox2 is significantly down-regulated in ALDH3A1 knock-down cells as compared to control cells (*n* = 3 independent experiments). MGMT methylated cell lines presented in green, MGMT unmethylated cell lines presented in orange. Data are presented as mean ± standard deviation (SD). ^*^p < 0.05, ^**^p < 0.01, ^***^p < 0.001, ^****^p < 0.0001 (unpaired student *t*-test). D: DMSO; T: TMZ; L: LGK974.

To test whether ALDH3A1 mediates the resistance to TMZ, we created glioma cells with genetically down-regulated ALDH3A1 expression (Figure 3A). Most efficient KD was achieved in cell lines GBM1, GBM10 and SF188 and therefore chosen for further experiments. In comparison to control cells we noticed that cells with down-regulated ALDH3A1 expression grew slower and were significantly more sensitive to TMZ (Figure 3B, 3C). In contrast, ALDH3A1 knock-down cells did not alter their sensitivity towards LGK974 and combination treatment with TMZ (Figure 4A).

### ALDH3A1 inhibition reduces *in vitro* clonogenicity and the expression of stem cell markers

**Figure 5:**
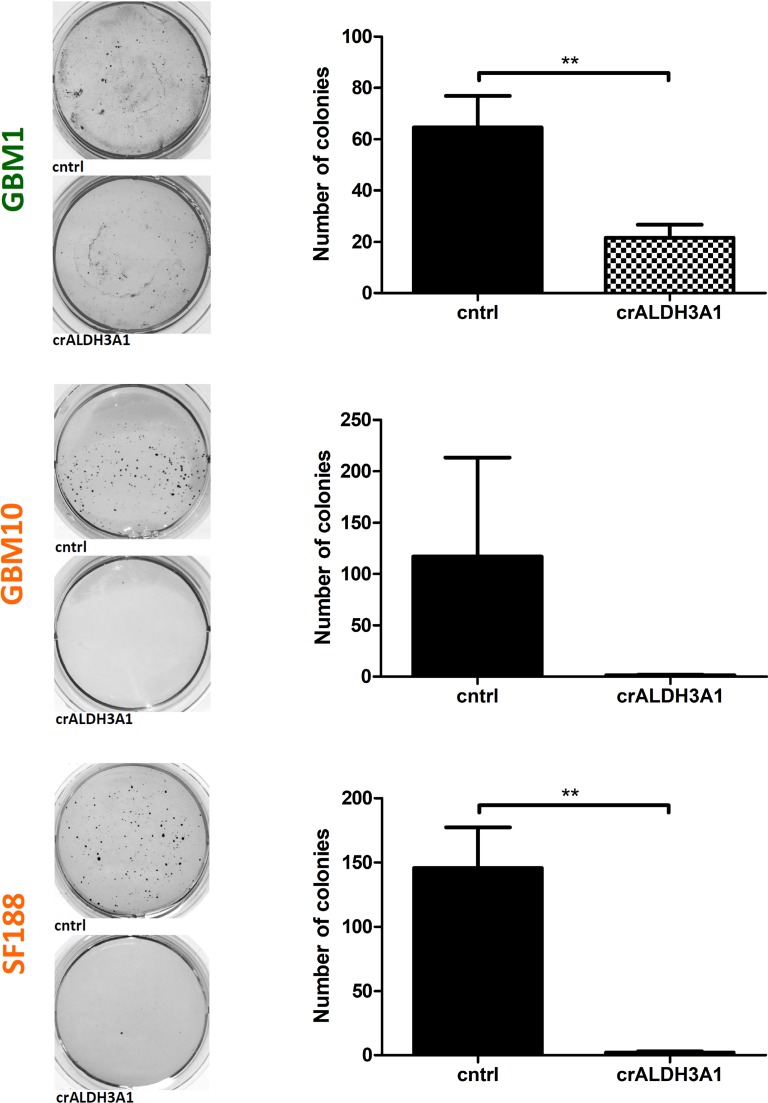
Clonogenicity is reduced in *ALDH3A1* knock-down cells Representative pictures of NBT stained colonies and quantifications of three colony forming assays are shown (*n* = 3 independent experiments). ^**^p < 0.01.

To reveal effect of ALDH3A1 knock-down on glioblastoma stem-like cells, we tested the expression of several established stem cell genes in our genetically modified cell models. ALDH3A1 GBM10 knock-down cells showed reduced CD133 protein levels, whereas in GBM1 and SF188 we noticed a tendency of reduced C133 expression (Figure 4B). Of note, in cell lines when blocking ALDH3A1 we observed a significant decrease in mRNA expression of *Nestin* and *Sox2* (Figure 4C) as compared to control cells. Additionally, cells with blocked ALDH3A1 caused a strong significantly reduced the total sphere formation capacity (Figure 5).

## DISCUSSION

Current therapeutic options for glioblastoma patients result in unsatisfying clinical outcomes. In particular, novel treatment options are highly needed for patients suffering from glioblastomas without *MGMT* promoter methylation who show limited benefit from TMZ chemotherapy. Our findings suggest that the enzyme ALDH3A1 might act as a therapeutic target whose inhibition sensitizes glioma cells to TMZ. Of note, ALDH3A1 blockade increased TMZ sensitivity independently of the *MGMT* promoter methylation status. Moreover, we identified Wnt signaling as an upstream regulator of this mechanism by showing that targeting Wnt pathway activity down-regulates the expression of ALDH3A1. This is of interest as Wnt signaling emerges as a therapeutic target in glioma stem-like cells [13]. Of note, in SF188, the only pediatric GBM model in our study, we observed significant differences in ALDH3A1 mRNA and protein levels in the context of drug treatment experiments. We speculate this may be a consequence of various post-transcriptional and epigenetic regulations as well as a possible negative protein-to-transcription feedback loop as previously described in large-scale glioblastoma datasets [18].

In addition to confirming recent findings that Wnt signaling promotes chemoresistance of glioblastoma cells [19], our data suggests that a possible mechanism of this association is the down regulation of ALDH3A1 expression in response to pathway inhibition. We chose ALDH3A1 as our target of interest since family members of the ALDH group are known to detoxify reactive aldehydes caused by treatment with alkylating chemotherapeutics [20, 21].

We consistently observed most synergistic anti-growth effects in lower dosages of TMZ and LGK974. This observation might be explained by the dose-effect curve for LGK974 indicating high effectiveness in low dosages. Given the particularly high synergistic effect observed when treating the cells with low drug concentrations, we hypothesize that adverse effects could be minimized and thereby favoring clinical applicability.

Additionally, our functional data suggest the utility of high ALDH3A1 expression as a putative diagnostic marker for stemness in glioma as indicated by reduced expression of CD133, Nestin and Sox2 as well as diminished clonogenicity in response to ALDH3A1 inhibition. Further correlative investigations in clinical datasets and *in vivo* models are needed to verify this hypothesis. Moreover, decreased overall cell viability following reduced ALDH3A1 expression could be due to the depletion of glioma cells with stem-like properties. However, additional functional studies are needed to comprehensively decipher the mechanistic background of interaction between stemness and cell growth in relation to ALDH3A1 expression and Wnt signaling activation.

The data reported in the present study is in concordance with observations in other solid tumors. In both preclinical and clinical studies it has been reported that high levels of ALDH3A1 promote chemoresistance towards various common anti-cancer drugs and could be correlated with poor clinical prognosis [15, 22–26]. Moreover, reports on other tumors outside the central nervous system suggest a positive correlation of ALDH3A1 expression and β-catenin signaling levels [26–29]. In head and neck squamous cell carcinoma, activation of ALDH3A1 increases chemoresistance against cisplatin whereas combining cisplatin with an ALDH inhibitor results in more pronounced cell viability reduction than treatment with each compound alone [30]. Other members of the ALDH family were already proposed to mediate therapy resistance in glioblastoma. ALDH1A3 was shown to increase resistance towards γ-radiation and to be up-regulated in high grade gliomas, whereas ALDH1A3 promoter-methylation correlated with longer survival time [31, 32]. Schäfer *et al.* identified ALDH1A1 to induce resistance towards TMZ in glioblastoma *in vitro* [33]. Importantly, we could not find any correlation between spontaneous ALDH3A1 mRNA expression and resistance levels to TMZ, suggesting that sensitivity to TMZ is only partly mediated through ALDH3A1. However, we provide evidence for ALDH3A1 inhibition to function as sensitizer to the glioblastoma standard of care chemotherapeutic agent.

The porcupine inhibitor LGK974 reduces Wnt signaling and decreased expression of ALDH3A1 mRNA and protein in glioma cells, thereby increasing their susceptibility to TMZ treatment. Regulation of *ALDH3A1* transcription by Wnt pathway activity may be mediated by increased TCF/LEF binding to the *ALDH3A1* gene promoter harboring TCF/LEF binding motifs (Supplementary Figure 3) [34]. However, this hypothesis requires further experimental proof. By comparing the results from wildtype cells with those from cells with genetically inhibited ALDH3A1 expression, it seems as if the ALDH3A1 knock-down is more efficient in reducing cell viability than pharmacological Wnt-inhibition. This result might be explained by the fact that LGK974 is a porcupine inhibitor and hence possibly not only affecting Wnt signaling but also further off-targets. Nevertheless, our results also suggest that the observed suppression of cellular growth upon Wnt blockade is mediated by suppression of ALDH3A1 for the most part, since LGK974 treatment has no effect on ALDH3A1 knock-down cells. Besides, we could not find a correlation between Wnt activation and ALDH3A1 expression in our cell lines, indicating that ALDH3A1 might not only be regulated through Wnt/β-catenin signaling but ALDH3A1 expression is also affected by other cellular dynamics.

Our findings also suggest that ALDH3A1 may be a promising therapeutic target for glioblastomas resistant to the standard of care treatment. The potential of ALDH3A1 as a therapeutic target with low adverse effects has been shown in *ALDH3A1* knockout mice [35]. Except for eye cataracts, a consequence of destruction of fiber cells that are dependent on ALDH3A1 to minimize oxygen damage, *ALDH3A1* knockout mice showed equal survival and growth as control animals. Of note, specific inhibitors of ALDH3A1 have been developed and shown to enhance the sensitivity of cancer cells to the alkylating agent cyclophosphamide [20, 22]. Our data also indicate that ALDH3A1 may regulate TMZ sensitivity in glioblastoma cells independently of the *MGMT* promoter methylation status. However, preclinical *in vivo* studies with drugs targeting ALDH3A1 are compulsory to further substantiate our *in vitro* findings and translate them into novel targeted treatment approach. Whereas ALDH expression serves as biological marker in solid tumors, we could not verify any prognostic value for ALDH3A1 in glioblastoma by searching several data bases (The Cancer Genome Atlas (TCGA) https://cancergenome.nih.gov/, The Cancer Imaging Archive (TCIA) [36], Murat *et al.* [37], Reifenberger *et al.* [38]).

In summary, our results reveal that ALDH3A1 expression in glioma cells can be modulated by Wnt pathway inhibition. Furthermore, we show that down-regulation of ALDH3A1 increases TMZ sensitivity and reduces stemness features in glioma cells *in vitro* independent of the *MGMT* promoter methylation status. These findings may have clinical significance by suggesting inhibition of ALDH3A1 as a potential strategy for increasing TMZ efficacy and particularly targeting the highly malignant subpopulation of stem-like glioma cells.

## MATERIALS AND METHODS

### Cell culture, pharmacological and radiation treatment

All four glioblastoma cell lines (GBM1, GBM10, JHH520, SF188) were cultivated in neurosphere medium containing 70% serum-free Dulbecco modified Eagle medium and 30% F12 (both Gibco BRL, Eggenstein, Germany), supplemented with 2% B27 (Gibco BRL), 20 ng/ml bovine fibroblast growth factor (Peprotech, Rocky Hill, NJ), 20 ng/ml human epidermal growth factor (Peprotech), 5ug/ml heparin (Sigma-Aldrich, St Louis, MO) and 1% Anti-Anti Penicillin Steptomycin Fungizone® mixture (Gibco). All cell lines were cultivated under standard cell culture conditions at 37°C temperature and 5% carbon dioxide. They were regularly tested for the absence of mycoplasma contamination using the PCR-based Mycoplasma Test Kit I/C from Promokine (Heidelberg, Germany). GBM1 was generously provided by A. Vescovi (Milan, Italy); GBM10 was provided by C. Eberhart [39]; JHH520 was provided by G. Riggins; the pediatric GBM cell line SF188 was provided by E. Raabe [40] (all Baltimore, USA). LGK974 (Selleckchem, Houston, USA) and temozolomide (Sigma-Aldrich, St. Louis, USA) were diluted in DMSO (Sigma- Aldrich)and stored in −20°C/−80°C. Both drugs were diluted in medium and added after each cell passage. Cells were passaged every second day and medium containing fresh drug was substituted. For radiation treatment, cells were exposed once to γ-radiation using a Gulmay RS225 X-ray system from X-Strahl (Camberley, UK).

### *MGMT* promoter methylation analysis

The methylation status of the *MGMT* promoter was determined by methylation-specific PCR as reported before [41]. The glioma cell line A172 served a positive control for *MGMT* promoter methylation whereas DNA extracted from peripheral blood leukocytes served as a negative control.

### Cell viability assays

Triplicates of 2000–5000 cells per well (depending on cell line) were plated on laminin-coated 96-well-plates in 100ul media for 6 days. The definition of maximal inhibitory concentration (IC50) of applied stimuli (LGK974, TMZ, γ-radiation) was performed through quantification of reduction of cellular viability using the CellTiter Blue™ Cell Viability Assay (Promega, Fitchburg, USA) as previously reported [14]. Fluorescence was measured after 2 hours incubation time using the Tecan Safire 2 Multiplate Reader (Tecan, Männedorf, Switzerland) at 560ex/590em. For IC50 definition we compared the effect on day 6 after treatment while changing medium supplemented with fresh drug every 2nd day. For analyzing combinatory effects, cells were treated with 5 different concentrations (0.25×, 0.5×, 1×, 2× and 0.125× or 4× IC50) of each drug or γ-radiation as mono or combination therapy. Viability measurements where performed as described above for the IC50 experiments. The combination index (CI) was calculated as described before [42] using the program CompuSyn (ComboSyn Inc., Paramus, NJ. 07652 USA) [16]. A CI < 1 refers to a synergistic, CI = 1 to an additive and CI > 1 to an antagonistic effect.

### Gene knock-down in glioblastoma cells using Crispr/Cas9- and shRNA-based approaches

Lentiviral particles of the third generation were generated for infecting cells as reported [43]. Cells with stable integration were selected using 2 μg/mL puromycin (Sigma-Aldrich). For *ALDH3A1* gene knock-down we used the lentiCRISPRv2 plasmid (Addgene plasmid # 52961) [44]. Oligonucleotides for guide RNAs were designed using the CRISPR design tool provided by the Zhang lab (http://crispr.mit.edu/). Oligonucleotides targeting GFP were used as a control. β-catenin knock-down was achieved by cloning shRNA into a pLKO.1 vector (Addgene plasmid # 1248) [45]. The RNA targeting sequences used are provided in the Supplementary Table 1B.

### Quantitative PCR and microarray-based gene expression analysis

RNA extraction (RNeasy Mini Kit, Qiagen) and cDNA synthesis (using M-MLV reverse transcriptase, Promega) were performed according to the manufacturer´s instructions. For the qPCR SsoAdvanced SYBR Green Supermix (BioRad) was used in a CFX Connect Thermocycler (BioRad), and the reaction was normalized to the housekeeping gene β2-microglobulin employing the ΔΔC_t_ method. Primer sequences are provided in Supplementary Table 1A. Transcriptome-wide expression analysis was performed on GBM1 cells after 72 h under drug treatment using Affymetrix GeneChip PrimeView Human Gene Expression Arrays (Affymetrix, Santa Clara, USA). Total RNA preparations were checked for RNA integrity by using the Agilent 2100 Bioanalyzer system (Agilent Technologies, Santa Clara, USA). All samples in this study showed high quality RNA Integrity Numbers (RIN = 10). RNA was further analysed by photometric Nanodrop measurement and quantified by fluorometric Qubit RNA assays (Life Technologies). Synthesis of cDNA and subsequent biotin labelling of cRNA was performed according to the manufacturers´ protocol (3′ IVT Plus Kit; Affymetrix Inc.). Briefly, 100 ng of total RNA were converted to cDNA, followed by *in vitro* transcription and biotin labelling of cRNA. After fragmentation, labelled cRNA was hybridized to Affymetrix PrimeView Human Gene Expression Microarrays for 16 h at 45°C, stained by streptavidin/phycoerythrin conjugate and scanned as described in the manufacturers´ protocol. Data analyses on Affymetrix CEL files were conducted with GeneSpring GX software (Vers. 12.5; Agilent Technologies). To further improve signal-to-noise ratio, a given probeset had to be expressed above background (i.e. fluorescence signal of a probe set was detected within the 20th and 100th percentiles of the raw signal distribution of a given array) in all four replicates in at least one of two, or both conditions to be subsequently analyzed in pairwise comparisons.

### Western blot analyses

Total proteins were extracted from glioma cells using RIPA Buffer as reported. Protein concentrations were determined in a Tecan Safire 2 Multiplate reader (Tecan) using the DC Protein Assay Kit (Biorad) due to the manufacturer´s instructions. Primary antibodies (ALDH3A1, 1/1000, abcam # ab76976; β-catenin, 1/1000, BD # 610153; α-Tubulin, 1/10000, Sigma # T9026; CD133, 1/100, Miltenyi Biotec # W6B3C1) were incubated overnight at 4°. Secondary antibodies (goat-anti-rabbit, IRDye800CW LI-COR # 926-32211; goat-anti-mouse, IRDye680RD LI-COR # 926-68070; goat anti-rabbit-HRP, Jackson Immuno Research # 111-035-144; all 1/10000) were incubated 1h at room temperature. All antibodies were diluted in blocking solution containing 5% milk powder in Tris-buffered saline with Tween20 (TBST). Signals were detected using a film based system by applying Super Signal West Pico Chemiluminescent Substrate (Thermo Scientific) or a luminescence based system in a LI-COR Odyssey CLx Imager (LI-COR). Densitometry was done using supplied software from LI-COR or ImageJ software.

### Reporter assay for measurement of Wnt/CTNNB1 activity

To detect canonical Wnt pathway activity we installed a stable transfection of our cells with a reporter construct containing seven TCF binding sides followed by a firefly luciferase cassette, as previously described [14]. Transfected cells were selected using 2 μg/ml puromycin (Sigma-Aldrich). For each measurement, cells were harvested and washed in PBS. According to the manufacturer's protocol the cells were lysed in Lysis Solution (Life Technologies # T1003). Luminescence readout was performed at 490 nm emission wavelength on a TriStar LB941 luminometer (Berthold Technologies, Bad Wildbach, Germany) and normalized to ß-galactosidase activity.

### Clonogenicity assay sigma-aldrich

For assessing clonogenic capacity of our cell lines we performed a colony formation assay in soft agarose as described previously [14]. Six-well plated were coated with a bottom layer consisting of 1.5 ml of 1% agarose (Life Technologies) and neurosphere media. On top a 2 ml layer consisting of 0.6% agarose containing 5000 cell/well was plated. It was covered with additional media (2 ml). After 3 weeks, 1 mg/ml 4-Nitro blue tetrazolium chloride (NBT) solution (Sigma-Aldrich) was added to stain the colonies overnight at 37°C. The experiments were quantified using Clono Counter software [46].

### Analysis of human tissue samples and published glioblastoma expression datasets

For prognostic associations of *ALDH3A1* mRNA expression in glioblastoma patients, we retrieved publically available data sets from *Reifenberger et al.* (GEO accession no. GSE53733).

### Statistical analyses

All cell biological experiments were done in at least three independent experiments and results are shown as mean ± standard deviation (SD). An unpaired student *t* test was performed with Prism version 4 (GraphPad Software Inc, La Jolla, CA) to calculate statistical significance and *p* < 0.05 was considered as significant. The correlation coefficient was also calculated using Prism. For transcriptome-wide expression analysis, probes within each probeset were summarized by Robust Multi-array Average (RMA) after quantile normalization of probe level signal intensities across all samples to reduce inter-array variability [47]. Input data pre-processing was concluded by baseline transformation to the median of all samples. To further improve signal-to-noise ratio, a given probeset had to be expressed above background (i.e. fluorescence signal of a probeset was detected within the 20th and 100th percentiles of the raw signal distribution of a given array) in all four replicates in at least one of two, or both conditions to be subsequently analysed in pairwise comparisons.

## SUPPLEMENTARY MATERIALS FIGURES AND TABLES



## Abbreviations

ALDH3A1: aldehyddehydrogenase 3A1
GSC: glioma stem cell
IC_50_: half maximal inhibitory concentration
IDH: isocitrate dehydrogenase
MGMT: O-6-methylguanine-DNA-methyltransferase
TCF: transcription factor
TMZ: temozolomide
Wnt: wingless
