# Supplementary material for: Inhibition of Wnt/beta-catenin signaling downregulates expression of aldehyde dehydrogenase isoform 3A1 (ALDH3A1) to reduce resistance against temozolomide in glioblastoma in vitro
Source: Oncotarget. 2018 Apr 27;9(32):22703–16. doi: 10.18632/oncotarget.25210 (PMC5978259; doi:10.18632/oncotarget.25210)
Supplement: Supplementary file 1 [file oncotarget-09-22703-s001.pdf]

Inhibition of Wnt/beta-catenin signaling downregulates expression of aldehyde dehydrogenase isoform 3A1 (ALDH3A1) to reduce resistance against temozolomide in glioblastoma *in vitro*

SUPPLEMENTARY MATERIALS

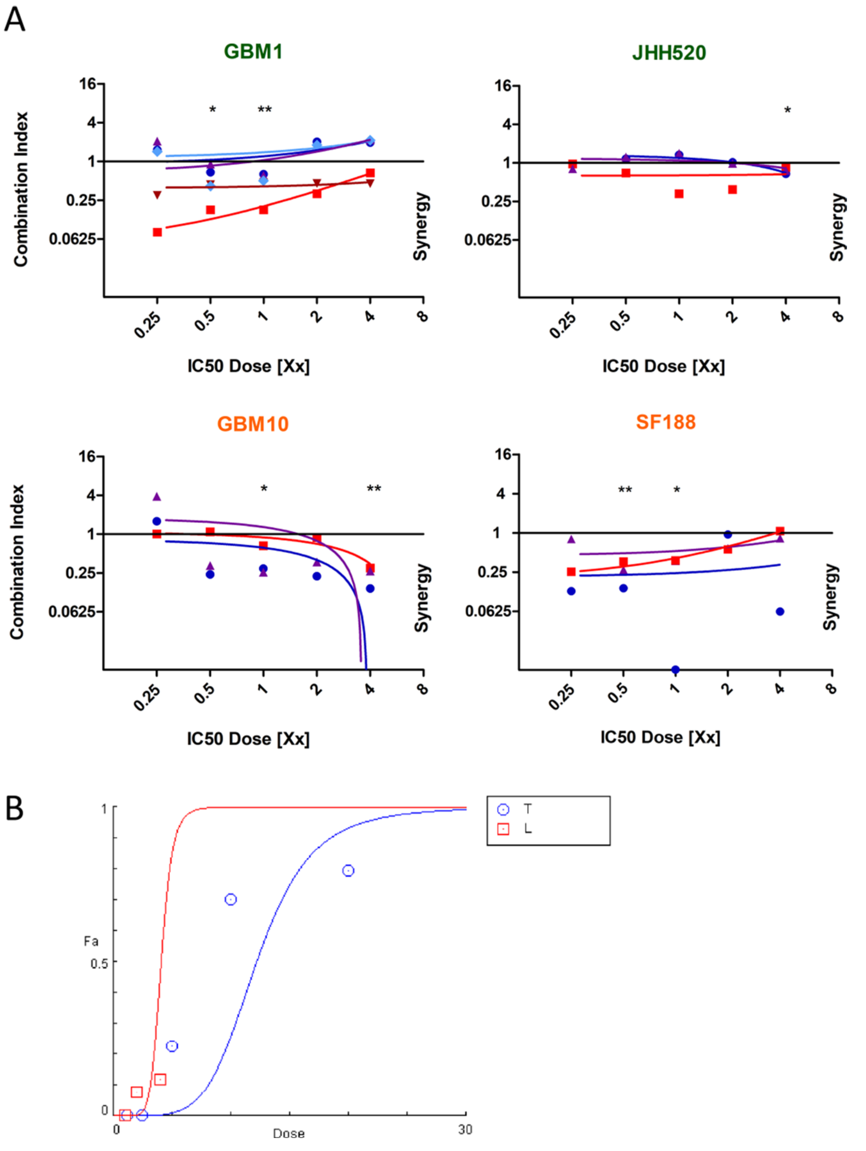

Supplementary Figure 1: (**A**) LGK974 acts synergistically in combination with γ-irradiation. The x-axis represents different multiples of the detected IC_{50} dose for each treatment and cell line. The axis of ordinates demonstrates the calculated combination index. The combination index is calculated based on the median-effect equation, taking each value from γ-irradiation, LGK974 single treatment and combination of both treatments for one specific dose into account. In one experiment, the combination index is calculated for five different doses and represented by one colored line. If the combination index is less than 1, both treatments act synergistically. If it is equal 1, both treatments act additively. If the combination index is more than 1, the effects are antagonistic. Each color represents one independent experiment (GBM1 n = 5 independent experiments; JHH520, GBM10 and SF188 n = 3 independent experiments). MGMT methylated cell lines presented in green, MGMT unmethylated cell lines presented in orange. (**B**) Dose-effect curve of TMZ and LGK974 in GBM1. Effect (Fa) is represented on the axis of ordinates; dose is represented on the x-axis on linear scales. D: DMSO; T: TMZ; I: γ-irradiation; L: LGK974.

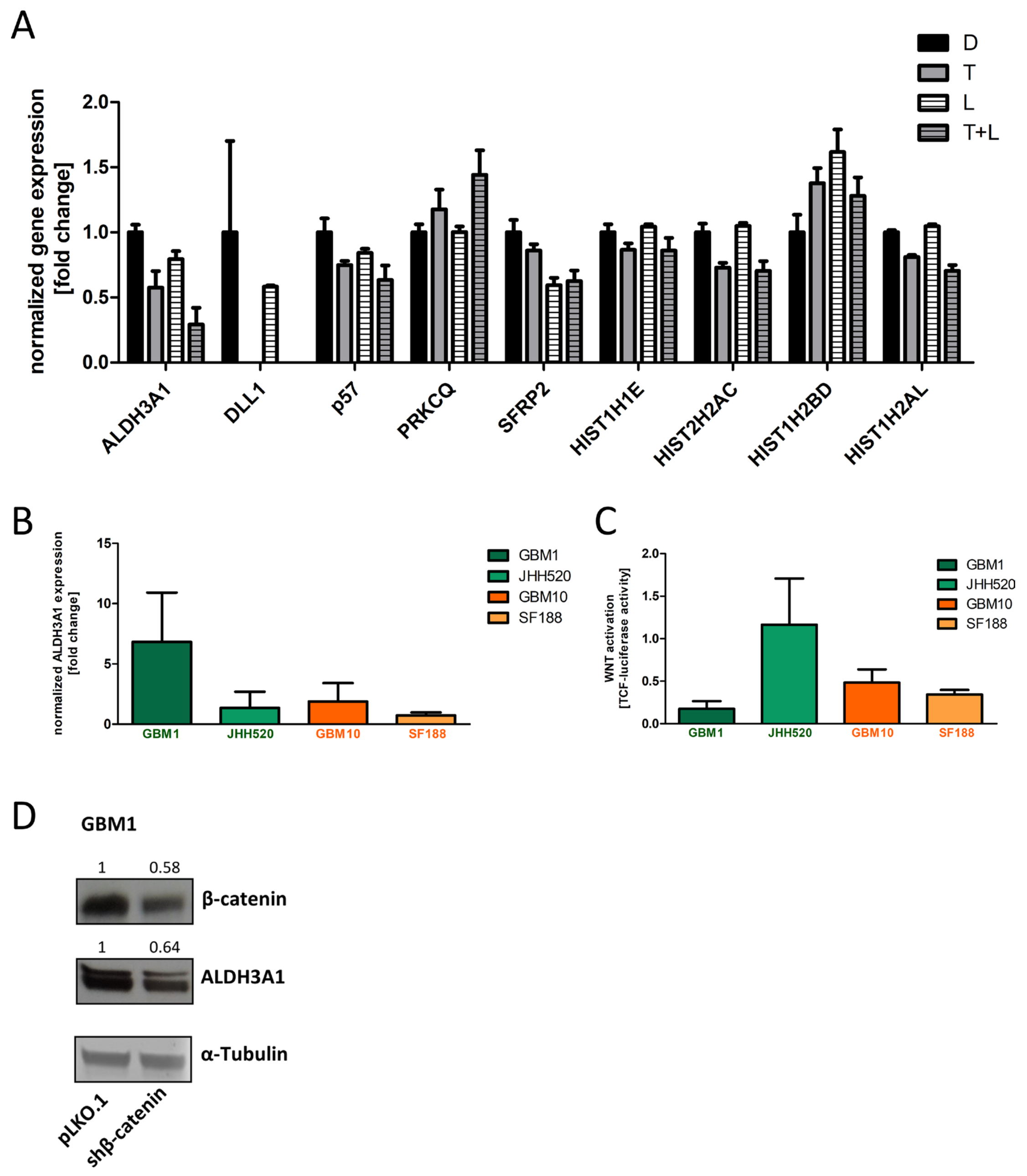

Supplementary Figure 2: (**A**) mRNA expression of 9 genes showing most pronounced differential expression in LGK974 and TMZ treated GBM1 cells as detected by microarray analysis. (**B**) Basal ALDH3A1 mRNA expression of all four cell lines. (**C**) Basal Wnt activation of all four cell lines assessed by T cell factor (TCF) luciferase reporters. (**D**) ALDH3A1 protein expression is reduced in GBM1 β-catenin-knock-down cells. pLKO.1 serves as the vector control. Data is presented as mean ± standard deviation (SD). D: DMSO; T: TMZ; L: LGK974.

```
   1 tttcatgtgt gatgagggtg cattccgtgt ccgttaagat ccaggacggg gcttcccaga
  61 ccccaagaga ctggcagtac caacccaagc gtgccctggc atgtcctcct ggcatgcccg
 121 ctgtgtccct tccaggatgg ccggggtgtg ggctggtctg cagaggctct gcagctgggg
 181 ctgcagccag gcggtgactc aggcaggttc cgcctgggct aggagggccg ggcattcctg
 241 ctgtgaggct gcccgtgcct aactcaccat gacttgcact ttgctgccat cctgcctggc
 301 aggctgactt agccaggatg gccctagtac ccacttcccc tccagccagc actcatgcct
 361 tctgccccgc taagatgccc tttgtcctga ctcggcagag gcttcctggg agccagggcc
 421 tgggcctggg agccacagct tcctgggagc aggaaagtcc aagggagatg ggtgaggac
 481 tggggcctct gcagcatctt aggggaggct ccccccttgct cgcactcacc tccgctggct
 541 gcaaacctgc agcttctctg acccaaggca ctatgactgg cagcagataa agagatgaag
 601 aagccccaga agtcctttcc tacaagtccc cagaaggtgt ggacacagtg gggaaattgc
 661 cccgggcaca gccagttctg ctgacttcat gcctctccaa agaggggctt tgtaacagta
 721 acagagggct ttgtaacaga ctgactgctc ttggctgggc acggtgactg gattacagct
 781 ctctgtggcc ttcaactcct gggctcaagc gatcctccca cctcagcttc cccgagtagc
 841 tgggactgca ggtgcgtgcc accacgccca gcagattttt acattttttg tagagatagg
 901 gtcttgcttg gtcacccagg ctggtctcaa actcctgacc tcaggtgatc tgcccgcctc
 961 ttcctcccaa agtgctggga ttacaggtgt gagtcaccgt gcctggctga acactaagtc
1021 gtattatttc tacctaaata tgtgtttgta cgcactaagc aagctctctt tattccccca
1081 cctccccacc cttcccagcc ctggtaacca ccgatctatt ctctatcttc atgagatcca
1141 cttgtttggc tcccacacat gagggagaac atgtggcatt tgtcttcctg agtcataaca
1201 cttttaacca acggggaggt ccctgagacc cactgcctaa ttttctcctt tctactctct
1261 cttttttttt tgagacaggg tctacctggt cactctgtcc cccaggctgg agtgcactgg
1321 catgattaca gctcactgaa gcctcgacct cctgggctca agtgatcctc ccgctcagcc
1381 tctcaagtag ctgggtgcat gccaccacgc ccagctaatt tttttttttt tttttttttg
1441 agatggagtc tcgctctgcc ctcatccaag ttggagtgca gtggcgcaat cttggctcac
1501 tgcaacctcc acctcccagg ttcaagcgat tctcctgcct cagcctcccg agtagctggg
1561 attacaggca tgcaccacca cggccagcta atttttgtat tttagtagag atggggtttt
1621 accatgttgg ccaggctggt cttgaactcc tgagctcaag tgatctgccc gcctcaacct
1681 cccaaagtgc tgggattaca ggcgtgagcc accgcaccca gcctccttcc ttttttttat
1741 tttttgtaga gatgagtctc tctatgttgc ccaggctcct tctactcgta taaagaaagt
1801 ggtttcccct cccaacaact atatctaatc taaatgttcc ttgaaattta agcaaactcg
1861 ctattcattc attcattcat tcattcaaca cttttttttgc tagctttggg tgaatttgct
1921 gaaaatcgtc ttgctaaatg attgcttttc catcagaggg atggttaggt gtagtgggct
1981 gcttccgtat cttcagaggt ctcccttgtg ccaggatatg gtgctgaaca aaagttgaga
2041 tctctgcctt tgtggacatt ccagtccaag gggaataag gagagacata gtcacacaaa
2101 caaagatatg aacgcaggtt gtgacaggtt cctggaagta aggaaggttt tatttgagag
2161 gtgagagagg tcagggaagc tgagatctga acatggataa gagttagtct ggtgcagagt
2221 ggaagaaaaa gtgtcccagg gagcaagaac agcatgtgca aaggccttgt ggtaggaaat
2281 tgcctggtta agtcaaggaa tgccagcaga ctggagtgca gtgagaatgg tgggaagtgg
2341 tgcgaagtga ggctggcaag gcagccagga gccaggccag gcaggggctc acaggccatg
2401 ggaggaagct tcctgacagc aagggggtgc cacccaagga tgtgaatcag gggagagaca
2461 tgctcaggtc cttatcgtcc gcatgtctct ctggcagcca cttggagaag gcagctgagc
2521 ctgactcaag gagagtaggt agagggcaag aacagtggcc tgggagagag agagtgagtg
2581 acagcccagg tgaggatgag gcattgggga tggagtatta ggacttgcaa agattaactc
2641 atgcttgcaa tcccagcact ttgggaggct gaggcgggaa gatgaattga gcccaggagt
2701 ttgagaccag cctgggcaac ataatgagac ccatctctac aaaacattta aaaattagcc
2761 aggcatggtg gtgggttcct gtagtcacaa ggtgggagga tcgcttgagc accggaattc
2821 aaagctgcag tgagccgtga tggcaccact gcactccagc ctgggcaaca gagcaacacc
2881 ctgtctcaaa aaaaaaaaaa aaaaaagaaa aaagaaaatc tgaaaagcaa gtatctgcaa
2941 gtttctagca caacccacac tcccacactt ttgccactac tgaaactata accaaaaccc
3001 aaacacgcaa acaaggattt tgttcttcgt agatttgaac tgcttttagg ctccttgctc
3061 tgacgcttcc accactactg ctgccacttt tgatgacacc cacaccacct aacctttcca
3121 ggatgccctc ggcccgccag gcactgttct ggtcacttcg tgtactcaat gctgtgttgc
3181 tggcttttgc tagttgcttc catggttctc ccaggggagg gccgctgcca caagaagtca
3241 gctccgaatg gccttcggcc ttgcattctg ccctctactc aagatccaaa ttcccaggag
3301 agtcagggag gcctcatggg gagcccaccc caacccatgg ctggcctgga gaaggcacct
3361 ggttgatcat ctcaccaagt aggaaggggt tgcctgcaaa gataatcagc tgctgccacc
3421 agaaaagggg agaggctggc acaggctcgg tcttctcccc tctcaggagg caccccactt
3481 tgcacctacc acccagtcca gcccctgcac aggtgcgagg tcctgcagat gcttctttct
3541 gctgtttctg gaatgtttcc cattctgtta gtcttatttg ggggtctcgt catttcttgc
3601 ctgcattgct ggaagctgca gcctttgggc tagacctttc tcacactgtg ccctgtccag
3661 ggggttctct aaaacaaaaa atagcagaca tttgtctgtc tgataatctc ctcagcgccc
3721 tgcaccccca cagccagcct ggcccacagg cctccgtgag cccccgcccc aggagagtca
3781 ttcacctcca cacctcctgc tggcaacagt ggacacatcg cactttttcg tgacataacg
3841 ttcccagcct ctgtgccttt gcttctgctc ttccagcccc cttgctgttc cttgcccaaa
3901 tgcttctcac tcagctcaga tgatgttccc tcccacggac tctcacccca gcccctctag
3961 catcctctgc ctacctggtt actacagtgt tcacattatt ttgaaatttc ctgtttattt
4021 tgtcagtcaa taaatattta ttgagcatct actataaatg ctgtatggag gttacagcag
4081 tgaacaaatc aaattctagc tgtcaggagt taagggacag gtagcaaaca agatataaac
4141 gctccagaga aaaaataatg gaggaagacg acagcattgt gctattttcc acagcacatt
4201 ggagcgtagc cgtgtgggtg gatgtagggg aagggggctc caggtggaga gaagcaggag
4261 ggccagagca catcctcagg actcgtaggc caggaccgga accttgtgtt ttcctcgaat
4321 gaaatgggaa gcagggaccc catgctggta agcagtctgc ctgcaggcgg gtgagggtgg
4381 cgggaagcgc ctggtgagag gggccgagag tctgttccac acctgcctcc tccccacagc
4441 gtggtgcaga gcaggagccc agcgaacatt gatcagacag cgtgggaacg gcttttccag
4501 actccttcct tcccaagcat ctctggtttt cggtgaccac acagaccctt ggccctcttc
4561 tcagctattc aacgagagag cagatttatg gattggagac ccccatcaaa agtccccagc
4621 ctgtggctcc ccagggttag gatctctgac tcaatggggc cctgtgcaca ctgggggatg
4681 tgaagggccc ggggcagcgg tggctggggg tgcgtttcgg gggagctgca tgctctcact
4741 gtggttcgtg gctgagccct gccgatctta tgtaaccaga gaactcagag aaccgcatct
4801 ggcagcggca agtctggaaa gctggaagag ctccatgcca ggctgaatca atcagcagcc
4861 cccacgccca gggcaaacat aggctctttt gaagattgga gatgtgccct gcccaagcct
4921 tcagaaacat tctcgcattt cagaagtgaa caaaagcaaa cagcccggga cctaatcccc
4981 aaaacctggg ctgtagggag
```

Supplementary Figure 3: Genomic sequence on chromosome 17 (nucleotides 19753433-19748433) with binding sides for TCF in der *ALDH3A1* promoter marked in blue. The sequence was retrieved from NCBI GenBank.

Supplementary Table 1: Primer sequences used for qPCR analyses (A) and oligonucleotides used as RNA-targeting sequences for CRISPR/Cas9-mediated knock-down of ALDH3A1

A

Primer	fwd	rev
β2-microgobulin	GTTGCTCCACAGGTAGCTCTAG	ACAAGCTTTGAGTGCAAGAGATTG
ALDH3A1	TGTTCTCCAGCAACGACAAG	CTGACCTTCAGGCCTTCATC
P57	GCTGAACGCCGAGGACCAGAACCG	CGGGCACCGAGTCGCTGTCCACTT
DLL1	TGGCGCAGGCATCGA	GGCGGCTGATGAGTCTTTCT
PRKCQ	CTCCCTTATATGGCCCCTGG	CTTCTGCGATGCCACTGTAC
SFRP2	TTCCCCAAGCACACTCCTAG	TACAAGATTCGGGTGGGCTT
HIST1H1E	CCAAGAAGAGCGCCAAGAAG	CGCCTTGGGTTTAACTGCTT
HIST2H2AC	GTGGCAAACAAGGAGGCAAG	GTCTTCTTGTTGTCCCGAGC
HIST1H2BD	ACCGGCACCTTGATCTTGTA	GGCTGGGGAGTAAAGAGTGT
HIST1H2AL	GACAACAAGAAGACCCGCAT	CTCGGTCTTCTTGGGCAGTA
Sox2	TGGACAGTTACGCGCACAT	CGAGTAGGACATGCTGTAGGT
Nestin	GGCGCACCTCAAGATGTCC	CTTGGGGTCCTGAAAGCTG

B

Oligo	fwd	rev
Control	CACCGGGTGAACCGCATCGAGCTGA	AAACTCAGCTCGATGCGGTTCACCC
crALDH3A1	CACCGTTCGACCATATCCTGTACAC	AAACGTGTACAGGATATGGTCGAAC
crALDH3A1 2nd	CACCGGGACACCCCCATTGATTACT	AAACAGTAATCAATGGGGGTGTCCC
